# Advancing working memory research through cortico-cortical transcranial magnetic stimulation

**DOI:** 10.3389/fnhum.2024.1504783

**Published:** 2024-12-09

**Authors:** Phivos Phylactou, Nikos Konstantinou, Edward F. Ester

**Affiliations:** ^1^Faculty of Health Sciences, School of Physical Therapy, University of Western Ontario, London, ON, Canada; ^2^The Gray Centre for Mobility and Activity, Parkwood Institute, London, ON, Canada; ^3^Faculty of Health Sciences, Department of Rehabilitation Sciences, Cyprus University of Technology, Limassol, Cyprus; ^4^Department of Psychology and Institute of Neuroscience, University of Nevada, Reno, NV, United States

**Keywords:** working memory, transcranial magnetic stimulation, ccPAS, paired associative stimulation, cortico-cortical, sensory recruitment

## Abstract

The neural underpinnings of working memory (WM) have been of continuous scientific interest for decades. As the understanding of WM progresses and new theories, such as the distributed view of WM, develop, the need to advance the methods used to study WM also arises. This perspective discusses how building from the state-of-the-art in the field of transcranial magnetic stimulation (TMS), and utilising cortico-cortical TMS, may pave the way for testing some of the predictions proposed by the distributed WM view. Further, after briefly discussing current barriers that need to be overcome for implementing cortico-cortical TMS for WM research, examples of how cortico-cortical TMS may be employed in the context of WM research are provided, guided by the ongoing debate on the sensory recruitment framework.

## Introduction

Working memory (WM) describes the process for temporarily maintaining information that is absent from the environment, in order to guide task-oriented behavior. For more than five decades (Fuster and Alexander, 1971; Fuster, 1973; Niki, 1974), the neural underpinnings of WM have been of continuous scientific interest. Throughout this time, the study of WM has shifted from a strictly modular view, which considers specific brain regions as either essential or not for WM [e.g., Xu (2017) and Chai and Abd Hamid et al. (2018)], toward a distributed view, which suggests that WM is a networked process involving various brain regions and neural mechanisms depending on task demands (Miller et al., 2018; Christophel et al., 2017; Lorenc and Sreenivasan, 2021).

However, limitations of the current methodological repertoire, such as the correlational nature of neuroimaging techniques and their application with assumptions under the modular view (see also *Transcranial Magnetic Stimulation and Working Memory*), do not enable the robust investigation of some of the predictions of the distributed WM view (Lorenc and Sreenivasan, 2021). As our understanding of the underlying neural architecture of WM progresses, the need to advance the methods for studying contemporary WM theories becomes necessary.

This perspective presents a brief overview of how WM theories developed from a modular toward a distributed view of WM, and how utilizing state-of-the-art cortico-cortical (dual-coil) transcranial magnetic stimulation (TMS) techniques (Tarasi et al., 2024) might enable us to test some of the proposed predictions of the contemporary distributed WM view (Christophel et al., 2017; Lorenc and Sreenivasan, 2021). Further, the perspective describes potential examples of how these cortico-cortical TMS techniques may be applied to study the ongoing debate surrounding the sensory recruitment framework of WM (Xu, 2017; Teng and Postle, 2021).

### From a modular to a distributed view of working memory

A typical WM task involves the presentation of a memory sample that needs to be remembered, followed by a delay period of a few seconds, during which the memory sample needs to be maintained in WM. After the delay period, a probe is presented to test a feature of some (or all) items of the maintained memory sample, through an ocular or motor response (e.g., a saccade or a button press). From as early as 50 years ago, non-human primate electrophysiological research employing such oculomotor delayed response tasks, showed that elevated neural activation, specific to the to-be-remembered stimulus, persisted through the WM delay period, thus linking the maintenance interval between the memory sample and the response (Fuster and Alexander, 1971; Fuster, 1973; Niki, 1974; Funahashi et al., 1989).

Following the primate electrophysiological findings, functional magnetic resonance imaging (fMRI) studies tested the principle of persistent neural activation during the WM delay period in the human brain. In addition to electrophysiological studies, fMRI research presented vast evidence in support of the roles of the prefrontal cortex (PFC) and the posterior parietal cortex (PPC) in WM (Fuster and Alexander, 1971; Fuster, 1973; Niki, 1974; Funahashi et al., 1989; Ester et al., 2015; Bettencourt and Xu, 2016). Due to the consistent evidence of sustained elevated neural spiking during the delay period, persistent neural activity was considered the main neural marker of WM representations (Constantinidis et al., 2018; Curtis and Sprague, 2021; Riley and Constantinidis, 2016), with the PFC and PPC viewed as the primary brain areas responsible for WM maintenance (Xu, 2017; Constantinidis et al., 2018; Xu, 2020).

However, in the late 2000s, with the introduction of advanced computational methods in fMRI analysis (e.g., machine learning classifiers), evidence emerged showcasing that WM representations can also be decoded from the visual cortex (Serences et al., 2009; Harrison and Tong, 2009). These findings, in conjunction with non-human primate electrophysiological findings (Supèr et al., 2001), gave rise to the sensory recruitment framework (Pasternak and Greenlee, 2005), which poses that the visual cortex is not only involved in perceiving visual information, but also in maintaining specific visual information during WM (e.g., low-level visual features, such as orientation).

Even though the sensory recruitment framework still remains debated to this day (Xu, 2017; Teng and Postle, 2021; Xu, 2020), the ability to decode WM representations outside the scope of persistent neural activity ignited another discussion in the literature. This discussion challenged the notion of persistent neural activity as the sole neural marker of WM maintenance (Masse et al., 2020; Sreenivasan et al., 2014; Lundqvist et al., 2018), proposing that additional or alternative mechanisms, such as synchronization of neural oscillations (Miller et al., 2018; Bhattacharya et al., 2022; Luo and Ester, 2024; Daume et al., 2024) and latent storage processes (Rose et al., 2016; Fulvio et al., 2024; Lorenc et al., 2021), are crucial for understanding the neural underpinnings of WM. These debates around the sensory recruitment framework and the role of persistent neural activity, in addition to a broader understanding of a networked view of cognition in the brain (Barack and Krakauer, 2021), have resulted in a shift of the foundations of WM research, from a strictly modular view toward a flexible, distributed view of WM.

Traditionally, WM research aimed to pinpoint ‘*where*’ in the brain WM representations are stored, based on identifying persistent neural activity during the WM delay. This approach adopts a modular view of the brain, assuming that a specific group of neurons in a specific brain region is solely responsible for a specific process (Barack and Krakauer, 2021). This results in a binary investigation for a given brain region, where it is viewed as either essential or not for WM maintenance, for example, based on whether persistent neural activity can be found in the region during the WM delay. Undoubtably, this modular view provided important findings for the neural architecture of WM (Chai and Abd Hamid et al., 2018), however, as our knowledge deepens, neuroscientists are starting to move beyond this modular view, as it is now generally accepted that cognition can be better understood if studied as a distributed process (Barack and Krakauer, 2021). Under this distributed view, WM is regarded as a flexible, networked process, which can recruit different mechanisms from across the brain, in order to support WM maintenance, depending on different task demands (e.g., priority, load, distractors, stimulus complexity)(Christophel et al., 2017; Lorenc and Sreenivasan, 2021; Teng and Postle, 2021; Lorenc et al., 2021). For example, when priority is introduced for certain items in WM, persistent activity may drop below the detectable threshold for unprioritized items (Iamshchinina et al., 2021), but can return once priority is restored (Masse et al., 2020; Rose et al., 2016). As such, contemporary WM theories, move beyond studying ‘*where*’ in the brain representations are maintained, towards understanding ‘*how*’ representations are distributed across the brain network responsible for successful WM maintenance (Christophel et al., 2017; Lorenc and Sreenivasan, 2021).

Despite the increased acceptance of the distributed WM view, many of its theoretical proposals still lack empirical evidence (e.g., Christophel et al., 2017 and Lorenc and Sreenivasan, 2021). Partly, this may be due to the limitations of the current neuroscientific tool repertoire, such as the correlational nature of neuroimaging methods. A powerful tool, which if employed rigorously enables causal investigations of brain and behavior is TMS (Pitcher et al., 2021). However, the current use of TMS in WM research exhibits specific shortcomings, since it still relies on the assumptions of a modular WM view. These shortcomings limit the capability of testing networked processes with TMS, as proposed by the distributed view of WM. Guided by the TMS state-of-the-art, we propose that the current limitations can be overcome with the employment of cortico-cortical TMS. Cortico-cortical TMS, introduces causal manipulations to cortico-cortical networks (contrary to targeting a single region), thus providing WM researchers with a tool capable of testing the flexibility of WM across the cortical network. In the following sections, we provide a brief description of how TMS is currently employed to study WM, present the limitations of the current approach, and introduce how these limitations can be overcome with the utilization of cortico-cortical TMS.

### Transcranial magnetic stimulation and working memory

TMS has been used extensively to study WM (Phylactou et al., 2022). TMS is a non-invasive technique, which uses a coil over the scalp that delivers strong magnetic pulses in a targeted brain region. If delivered with adequate intensity, TMS can result in action potentials in the targeted neural population, which in turn can instigate physiological responses such as muscle twitches (e.g., when induced over the motor cortex) or phosphenes (e.g., when induced over the early visual cortex; EVC)(Phylactou et al., 2023; Phylactou et al., 2024). In the study of behavior, TMS is thought to interfere with the regular neural activity of the targeted brain region, and therefore, if the targeted region is required for the successful execution of the behavior under study, then changes in the behavior will be evident due to TMS interference (Pitcher et al., 2021). In the context of WM, TMS can be delivered, for example, over the EVC, the PPC, or the PFC during the delay period of a WM task, and in return the effects of targeted TMS on WM performance can be measured (Rose et al., 2016; Fulvio et al., 2024; Phylactou et al., 2022; van de Ven and Sack, 2013; Rademaker et al., 2017; Phylactou et al., 2023; Dake and Curtis, 2024).

Even though a thorough TMS study design may enable causal brain-behavior investigations (Pitcher et al., 2021; Bergmann TO and Hartwigsen, 2021) the current use of TMS for studying WM suffers from limitations, because its use rests heavily on the assumptions of a modular WM view. Put simply, the main question of past WM TMS studies was focused on whether a brain region is ‘essential or not’ for WM maintenance (Xu, 2017; Phylactou et al., 2022). Yet, it is possible that measurable effects in WM performance due to TMS, may not be directly attributed to the targeted brain region [e.g., diaschisis; Garcia et al. (2020)], hence, the possibility that any of the behavioral effects are a result of indirect brain interference cannot be ruled out.

For example, studies employing EVC TMS during WM maintenance, may provide evidence for a drop in WM performance (Rademaker et al., 2017; Phylactou et al., 2023), attributing the behavioral effect to interference with visual cortex activity. Such findings can be taken as support for the sensory recruitment framework on the basis that TMS interfered with EVC neural processes responsible for WM maintenance. Conversely, an alternative explanation of such findings may be that TMS did not directly interfere with EVC WM representations *per se*, but rather, EVC TMS resulted in indirect effects on WM, by interfering with back-projections from the IPS, where the representations might actually be maintained (see Xu, 2017 and Phylactou et al., 2022). Similarly, under the distributed WM view, different brain regions or storage mechanisms may be flexibly recruited or recruited in parallel depending on task demands or the behavioral context (Lorenc and Sreenivasan, 2021; Lorenc et al., 2021; Teng and Postle, 2024). As such, targeting a specific brain region with TMS, might not result in measurable differences in WM performance, not necessarily because the targeted brain region is not involved in the maintenance process, but because parallel coding in a different region (e.g., intraparietal sulcus; IPS) maintains the WM representation (Teng and Postle, 2024). To overcome these limitations, WM research may benefit from adapting state-of-the-art TMS techniques, such as cortico-cortical TMS, as discussed next.

### Cortico-cortical transcranial magnetic stimulation

**Figure 1:**
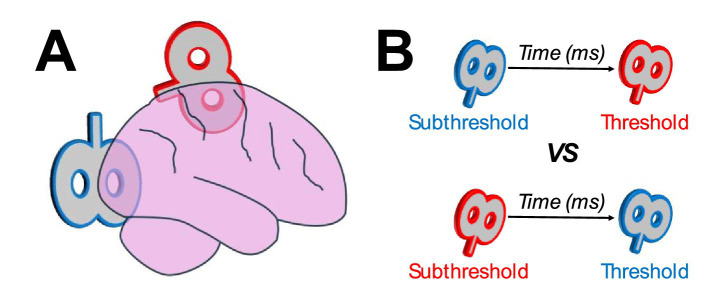
(**A**) Example dual-coil design of cortico-cortical transcranial magnetic stimulation targeting occipitoparietal networks. One coil targets occipital regions (blue) and another targets parietal regions (red). (**B**) Examples of stimulation protocols, with a weaker (subthreshold intensity) pulse in one region preceding a stronger (threshold intensity) pulse in another region. Based on the principles of spike-timing dependent plasticity, interconnected networks can be strengthened when the initial, weaker, pulse targets a lower-level region, followed by a stronger pulse in a higher-level region (top), or weakened when the initial pulse targets the higher-level region, followed by the lower-level region (bottom).

Cortico-cortical TMS uses a dual-coil design to simultaneously target two interconnected brain regions (Figure 1A). The theoretical foundation behind employing dual-coil TMS to target cortico-cortical networks is based on the principles of spike-timing dependent plasticity (STDP). According to STDP, associative pre-synaptic and post-synaptic activations are strengthened, depending on the temporal order and temporal difference of the pre-synaptic and post-synaptic spiking across the cortico-cortical network (Koch et al., 2013; Caporale and Dan, 2008; Markram et al., 2011). As such, interconnected networks can be primed through an initial weaker TMS pulse on a lower-level cortical region, followed by a second stronger pulse on a higher-level cortical region (Figure 1B, top). This priming paradigm can strengthen the connection across the network, while a reversal of this paradigm (weaker pulse on higher-level region first, followed by a stronger pulse in lower-level region; Figure 1B, bottom) can hinder the network’s connectivity (or have no effect on the network).

A dual-coil TMS protocol applied repeatedly over a cortico-cortical network (typically 90 paired pulses in total), is referred to as cortico-cortical paired associative stimulation (ccPAS). Even though ccPAS research has mainly focused on motor behavior (Trajkovic et al., 2023; Bevacqua et al., 2024; Turrini et al., 2023; Sel et al., 2021; Chiappini et al., 2024), recent work studying cortico-cortical visual networks and their role in cognition using ccPAS, has also emerged (Tarasi et al., 2024). For example, a recent study employed ccPAS and demonstrated that perceptual sensitivity and metacognitive decisions rely on distinct networks, with the former being improved only through ccPAS over V5/MT+ to V1 and the latter only through ccPAS over IPS to V1 (Luzio et al., 2022).

In detail, Luzio et al. (2022), asked participants to perform a motion discrimination task, which also included confidence ratings of the participants’ discrimination response, before, immediately after, and 30 min after a 15-min delivery of 90 paired pulses of ccPAS. Of note, ccPAS protocol effects are typically considered to last up to 60 min after their application and with some findings suggesting that they may peak at approximately 30 min (Wolters et al., 2005; Chao et al., 2015; Stefan et al., 2000). In three groups of participants, a different ccPAS protocol was delivered: (i) from V5/MT+ to V1 with a 20 ms interstimulus interval (ISI) between the two pulses, (ii) from IPS to V1 with a 30 ms ISI, or (iii) from IPS to V1 with 0 ms ISI (i.e., concurrent IPS and V1 stimulation). The 20 ms ISI in the V5/MT+ to V1 condition (Pascual-Leone and Walsh, 2001; Silvanto et al., 2005) and the 30 ms ISI IPS to V1 condition (Silvanto et al., 2009; Parks et al., 2015), reflected the timing required for V5/MT+ and IPS to exert physiological responses to V1, respectively. The temporal difference of the ccPAS paired pulses is in accordance with STDP principles, which suggest that in order to strengthen network activations, pre-synaptic activations should precede post-synaptic ones. The 0 ms ISI protocol served as a control protocol, since the simultaneous stimulation disregards the temporal difference required as per STDP principles. The authors reported that motion discrimination performance was only improved during V5/MT+ to V1 ccPAS, whereas confidence increased only during IPS to V1 ccPAS, irrespective of the timing (whether immediately after or 30 min after ccPAS). No changes to either discrimination nor confidence where found in the control ccPAS protocol.

Such studies, can pave the way for adapting ccPAS paradigms, enabling WM researchers to causally study different cortico-cortical pathways, in accordance with the predictions of a distributed WM view. However, to translate the current ccPAS state-of-the-art to WM research, some barriers need to be overcome to facilitate its successful and rigorous implementation. Some of these barriers and how they can potentially be addressed are discussed next.

#### Barriers and future directions

The implementation of cortico-cortical TMS, requires addressing some barriers, such as financial. For example, researchers may be required to purchase equipment, such as additional coils or TMS machines and synchronization apparatus. Moreover, to apply cortico-cortical TMS rigorously, researchers may need to perform anatomical brain scans to facilitate neuronavigated coil targeting.

Beyond the practical barriers, the successful translation of cortico-cortical TMS paradigms to WM research, currently relies on overcoming some knowledge limitations about the temporal dynamics of specific cortico-cortical networks. In detail, as proposed by STDP principles (Koch et al., 2013; Caporale and Dan, 2008; Markram et al., 2011), the temporal order and timing between the activation of pre-synaptic and post-synaptic neurons are vital for the successful manipulation of the targeted network. This is also reflected in TMS studies, where stimulation effects are only evident when ccPAS is tailored to the temporal dynamics of the targeted network (Trajkovic et al., 2023; Bevacqua et al., 2024; Turrini et al., 2023; Sel et al., 2021; Chiappini et al., 2024; Luzio et al., 2022; Hernandez-Pavon et al., 2023). Hence, to successfully study potential networks and their involvement in WM maintenance, it is important to identify the temporal characteristics of the targeted network.

Robust controls are also needed to draw accurate inferences from dual-TMS experiments. Since this approach entails delivering two TMS pulses with different temporal and intensity profiles to two different brain areas, one possibility is that any effects observed are due to interference from either of the two TMS pulses. For example, in a hypothetical experiment employing ccPAS to the IPS-V1 cortico-cortical network during WM maintenance, it is possible that a potential performance decrease following ccPAS, is caused solely because of TMS to V1 (or to IPS), and not due to the dual IPS-V1 ccPAS *per se*. To control for this possibility, multiple control conditions should be employed, as generally proposed when conducting TMS studies (Pitcher et al., 2021; Bergmann and Hartwigsen, 2021). For cortico-cortical TMS, these control conditions can comprise sham stimulation conditions and manipulations of the temporal order or temporal difference between the paired stimulations (Hernandez-Pavon et al., 2023). Additionally, single coil TMS conditions may be used as control conditions, to enable comparisons between interference to one cortical region (e.g., TMS only to V1 or IPS) versus the interference to the cortico-cortical network (ccPAS to IPS-V1).

Earlier studies have used ccPAS to study the premotor-motor network, and findings from this literature may provide a useful launch point for ccPAS studies of WM (Trajkovic et al., 2023; Bevacqua et al., 2024; Turrini et al., 2023; Sel et al., 2021; Chiappini et al., 2024). Evidence from these earlier studies has shown that when the low-level (premotor) region is stimulated first, ccPAS increases the connectivity of the network, as reflected through increased corticospinal excitability (Bevacqua et al., 2024; Turrini et al., 2023; Chiappini et al., 2024), and increased alpha, beta, and theta rhythms as reflected through scalp electroencephalography (EEG) (Trajkovic et al., 2023; Sel et al., 2021). When the high-level (motor) region is stimulated first, ccPAS either decreases the connectivity of the network (Trajkovic et al., 2023; Bevacqua et al., 2024; Sel et al., 2021) or has no evident effect compared to sham stimulation (Chiappini et al., 2024). Moreover, findings indicate that ccPAS effects are distinct from single-site TMS (Wang et al., 2022) and specific to the target cortico-cortical network (Hernandez-Pavon et al., 2023). In the context of WM, the groundwork may require testing the effects of various ccPAS temporal window profiles on WM performance, to identify the ideal temporal characteristics of a cortico-cortical network of interest (e.g., IPS-V1). Additionally, the temporal dynamics of potential WM networks of interest, may be informed through WM studies that utilize technologies with high temporal specificity, such as EEG (Dake and Curtis, 2024; Fulvio et al., 2024).

On the basis of current ccPAS evidence and focusing on the proposals of the distributed view of WM, the following section presents potential examples of how cortico-cortical TMS paradigms for WM research may come into fruition.

#### Cortico-cortical transcranial magnetic stimulation and sensory recruitment

The sensory recruitment framework poses that WM recruits the EVC (e.g., V1, V2, V5/MT+) for maintaining representations comprising low-level visual features, such as orientation, contrast, and motion direction (Serences et al., 2009; Harrison and Tong, 2009; Supèr et al., 2001; Pasternak and Greenlee, 2005; Phylactou et al., 2023; Konstantinou et al., 2012). The sensory recruitment framework has been a topic of ongoing debate (Xu, 2017; Teng and Postle, 2021; Xu, 2020; Gayet et al., 2018), mainly due to the failure of decoding EVC activity during WM maintenance under distraction in some fMRI studies (Bettencourt and Xu, 2016). To shed light on the debate, many researchers relied on TMS to provide causal evidence for EVC’s role in WM maintenance. However, findings from these earlier TMS studies are mixed and often controversial (Phylactou et al., 2022). At least in part, these mixed TMS findings, may be attributed to the current design of TMS experiments, which have been guided mainly by the traditional modular view of WM, resulting in two main limitations: (i) the focus on the essentiality of the EVC in WM instead of its role in supporting a networked process, and (ii) the failure to consider potential indirect effects of TMS interference (e.g., with IPS). Cortico-cortical TMS can overcome these limitations, and in accordance with more contemporary theories of WM, it can provide further insight into the neural underpinnings of WM.

**Figure 2:**
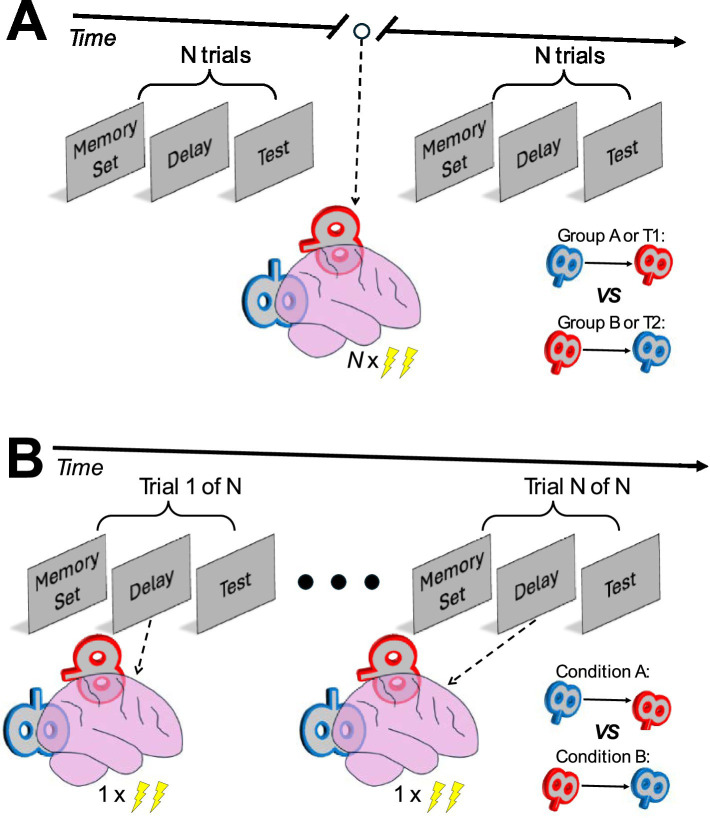
Examples of potential cortico-cortical transcranial magnetic stimulation (TMS) study designs to investigate the debated sensory recruitment framework. **(A)** Pre-/post- design where cortico-cortical paired associative stimulation is applied between blocks of working memory task trials. **(B)** Experimental design where different conditions of cortico-cortical TMS are applied during the delay of each working memory task trial.

One of the strongest arguments against the sensory recruitment framework, suggests that decoded activity in the EVC may not reflect WM maintenance *per se*, but instead, back-projections from higher-order brain areas, such as PFC or IPS (Xu, 2017). Using cortico-cortical TMS, IPS-EVC back-projections can be experimentally manipulated (Luzio et al., 2022), thus enabling a plausible design for a causal investigation of this argument. For example, WM performance can be compared before and after strengthening or weakening IPS-EVC back-projections using ccPAS (Figure 2A). Similar paradigms can be conceptualized, to study other potential cortico-cortical networks that may have a functional role in WM. For example, recent work demonstrated that feedforward and feedback processes, reflected through synchronized neural oscillations, link visual and frontal regions during WM guided behavior (Luo and Ester, 2024). As such, similar to targeting IPS-EVC networks, the paradigm can be adapted to target occipito-frontal networks.

Moreover, cortico-cortical TMS may be adapted in a manner that enables trial-by-trial experimental manipulations (Figure 2B). Though, and to the best of our knowledge, so far, cortico-cortical TMS has not been applied using a trial-by-trial design. If successful, trial-by-trial cortico-cortical TMS will allow testing of numerous manipulations within a single task, enabling randomization and control of various confounders (e.g., order effects, practice effects, etc.), potentially resulting in robust causal investigations of cortico-cortical networks. Nevertheless, due to limited prior work, whether a trial-by-trial cortico-cortical TMS design can be successfully implemented, remains an open avenue for future studies to explore.

Using a trial-by-trial design, different stimulation conditions can be employed during a WM task, thus facilitating manipulations leading to further insight about the targeted cortico-cortical networks. As an example, if studying sensory recruitment, switching the direction of the paired stimulation (e.g., from IPS-EVC to EVC-IPS) and comparing with rigorous control conditions (e.g., sham stimulation, or stimulating only one of the two coils), it may be possible to understand whether TMS effects targeted at the EVC result in direct or indirect effects on WM performance. Similarly, by introducing trial-by-trial task manipulations, it might be possible to study the flexibility of WM, for example, through manipulations that encourage participants to assign different levels of priority or importance to different stimuli held in WM (e.g., 32) or similarly, through manipulations that encourage participants to prioritize one feature of a set of stored objects (e.g., color) over another (e.g., shape) (e.g., Teng and Postle, 2021). In detail, previous studies found that activity patterns in the EVC and IPS are distinct for prioritized and unprioritized items held in WM (Rose et al., 2016; Iamshchinina et al., 2021). Considering that it is possible that a network process is involved in WM prioritization (e.g., Fulvio et al., 2024), an adaptation of cortico-cortical TMS may reveal distinct networks that support the maintenance of prioritized and unprioritized items. Further, WM representations may reveal behaviorally relevant effects only for specific features (Teng and Postle, 2024). For example, recent work (Teng and Postle, 2024) revealed that IPS may hold WM representations of multiple features (e.g., content and context), whereas EVC may maintain only specific features (e.g., content). Hence, cortico-cortical TMS could be employed to test parallel representation coding in the EVC-IPS network across different behavioral contexts. As such, trial-wise application of cortico-cortical TMS could reveal causal evidence for the roles of different networks in WM storage.

## Discussion

As our understanding of the neural underpinnings of WM progresses, the need to develop robust methodologies to test contemporary theories arises. This perspective showcases how adapting developments in the field of TMS, specifically cortico-cortical TMS, offer an avenue for the advancement of WM research. To illustrate how cortico-cortical TMS can advance WM research, we describe how it can be utilized to test one of the main arguments against the sensory recruitment framework; specifically, whether the role of EVC in WM maintenance is direct or it reflects back-projections from IPS. Despite current barriers, cortico-cortical TMS may provide an important tool in the methodological repertoire of WM researchers, which is aligned with the predictions of the distributed view of WM. By investing in the groundwork required to overcome current barriers, cortico-cortical TMS can facilitate the causal investigation of cortico-cortical networks, that will not only benefit the field of WM, but cognitive neuroscience in general.

## Data Availability

The original contributions presented in the study are included in the article/supplementary material, further inquiries can be directed to the corresponding author.
